# Effects of warming and nitrogen addition on soil fungal and bacterial community structures in a temperate meadow

**DOI:** 10.3389/fmicb.2023.1231442

**Published:** 2023-07-12

**Authors:** Ming Jiang, Yibo Tian, Rui Guo, Shuying Li, Jixun Guo, Tao Zhang

**Affiliations:** ^1^College of Life Science and Technology, Mudanjiang Normal University, Mudanjiang, China; ^2^Key Laboratory of Vegetation Ecology, Ministry of Education, Jilin Songnen Grassland Ecosystem National Observation and Research Station, Changchun, China; ^3^Institute of Environment and Sustainable Development in Agriculture, Chinese Academy of Agricultural Sciences, Key Laboratory of Dryland Agriculture, Ministry of Agriculture, Beijing, China; ^4^Forestry and Grassland Bureau of Aohan Banner, Chifeng, China

**Keywords:** combined effect, elevated temperature, eutrophication, soil microbial community, temperate steppe

## Abstract

Soil microbial communities have been influenced by global changes, which might negatively regulate aboveground communities and affect nutrient resource cycling. However, the influence of warming and nitrogen (N) addition and their combined effects on soil microbial community composition and structure are still not well understood. To explore the effect of warming and N addition on the composition and structure of soil microbial communities, a five-year field experiment was conducted in a temperate meadow. We examined the responses of soil fungal and bacterial community compositions and structures to warming and N addition using ITS gene and 16S rRNA gene MiSeq sequencing methods, respectively. Warming and N addition not only increased the diversity of soil fungal species but also affected the soil fungal community structure. Warming and N addition caused significant declines in soil bacterial richness but had few impacts on bacterial community structure. The changes in plant species richness affected the soil fungal community structure, while the changes in plant cover also affected the bacterial community structure. The response of the soil bacterial community structure to warming and N addition was lower than that of the fungal community structure. Our results highlight that the influence of global changes on soil fungal and bacterial community structures might be different, and which also might be determined, to some extent, by plant community, soil physicochemical properties, and climate characteristics at the regional scale.

## 1. Introduction

It is well known that soil microorganisms are the most active components in belowground ecosystems and play key roles in many ecosystem processes. Soil microorganisms can influence nutrient cycling (e.g., nitrogen and phosphorus), and decrease greenhouse emissions (e.g., CO_2_, CH_4_, and N_2_O) (Bender et al., 2015). Soil microorganisms can also accelerate litter decomposition (Kang et al., 2020), and determine plant community structure and net primary productivity (van der Heijden et al., 2008; Wagg et al., 2014). However, the composition of soil microbial communities has been influenced by global changes, such as elevated temperature (Xiong et al., 2014; Che et al., 2019), drought (de Oliveira et al., 2020), and eutrophication (Yan et al., 2022), which affect the ecological functions of soil microorganisms. In addition, changes in soil microbial structure can indirectly lead to global changes.

Several studies have found that warming has an impact on the community structure of soil microbes. For example, warming altered the soil fungal community structure in a Tibetan alpine meadow (Che et al., 2019; Zhang et al., 2020), had few effects on the community composition of soil fungi in a tropical grassland (de Oliveira et al., 2020), and increased the relative abundance of actinomycetes (Xiong et al., 2014). However, other studies have found that warming has not induced strong shifts in the soil fungal community in an alpine grassland (Deltedesco et al., 2020), and short-term warming has not altered the soil fungal community structure in permafrost and tallgrass prairies (Penton et al., 2013). Moreover, many studies have found that warming has altered the soil bacterial community structure and composition (Rui et al., 2015; Guo et al., 2019) and has increased the soil bacterial α-diversity in an alpine meadow (Li et al., 2016). Warming has increased the relative abundance of Chloroflexi in surface soil (0–10 cm) but reduced the relative abundance of Firmicutes at 10–20 cm (Zi et al., 2018). However, some other studies have found that short-term (1 year) warming has not altered the soil bacterial community structure in an alpine grassland (Zhang T, et al., 2016). These different responses illustrate that warming effects on soil microbial communities are not uniform and are determined by the types of ecosystems and the durations of warming.

Anthropogenic nitrogen (N) deposition has substantially increased in the last several decades. N deposition not only causes species richness losses (Bobbink et al., 2010) but also alters the structure of soil microbial communities (Ramirez et al., 2012; Zhou et al., 2017); for instance, studies have shown that the addition of N reduces the soil fungal biomass but increases bacterial growth (Zhou et al., 2017) and decreases soil bacterial richness (Wang et al., 2018). Recent research shows that combined organic and mineral fertilization increased microbial diversity (Wu et al., 2021). The latest meta-analysis results found that N addition significantly decreased the richness and Shannon index but increased the beta diversity of a soil microbial community (Zhou et al., 2020). However, some studies have found that N addition reduces soil fungal richness but not soil bacterial richness, indicating that the effect of N addition on microbial diversity is uncertain (Yuan et al., 2020). Moreover, changes in the soil microbial community are sometimes associated with the plant community (Ma et al., 2018), and the soil quality and plant community influenced by N addition play a vital role in the soil microbial community composition. However, how N addition alters a soil microbial community by affecting plant community composition is still not well understood.

The Songnen meadow located in the eastern Eurasian grassland is a typical temperate meadow and the largest temperate meadow in China. This meadow has been simultaneously influenced by elevated temperature and N deposition. Our previous results found that warming and N addition have significantly altered plant community structure, soil nutrient stoichiometry, and soil enzyme activities (Gong et al., 2015; Zhang et al., 2015; Mei et al., 2019). Few studies have focused on the effects of the addition of N and warming in temperate forests on the soil microbial community (Ma et al., 2018) or alpine meadows (Zhang et al., 2020), and the effects of warming and N addition on the soil microbial community in temperate meadow ecosystems are still not very clear. To explore the effects of warming and N addition on soil microbial community composition and to understand how warming and N addition affect soil microbial community structure by affecting plant community composition, a five-year field experiment was conducted to simulate warming and N addition in the Songnen meadow. We hypothesized that (1) warming and N addition would decrease soil fungal and bacterial richness and would alter soil fungal and bacterial community compositions because of the decline in soil moisture and the low N status in the grassland ecosystem; (2) the responses of the soil bacterial and fungal communities to warming and N addition would be asynchronous because of the difference in the strength of their symbiotic relationship with plants; and (3) warming and N addition would combinedly affect soil fungal and bacterial community compositions because warming could affect the process of N mineralization and availability.

## 2. Materials and methods

### 2.1. Study site

This study was conducted in the Songnen meadow (44°45′N, 123°45′E) in western Jilin Province, northeastern China. The mean annual precipitation is approximately 400 mm, of which nearly 75% of which falls between May and September. The average annual temperature is 5.0°C. The soil in the Songnen meadow is typical soda-saline type soil (Zhang T, et al., 2016), with a high pH of 9.0 and low organic matter content of approximately 3%. Global land surface temperature in the first two decades of the 21st century (2001–2020) was 1.59°C higher than 1850–1900 (Lee et al., 2023) and the total N input from precipitation has increased (Zhao et al., 2019).

### 2.2. Experimental design

**Figure 1:**
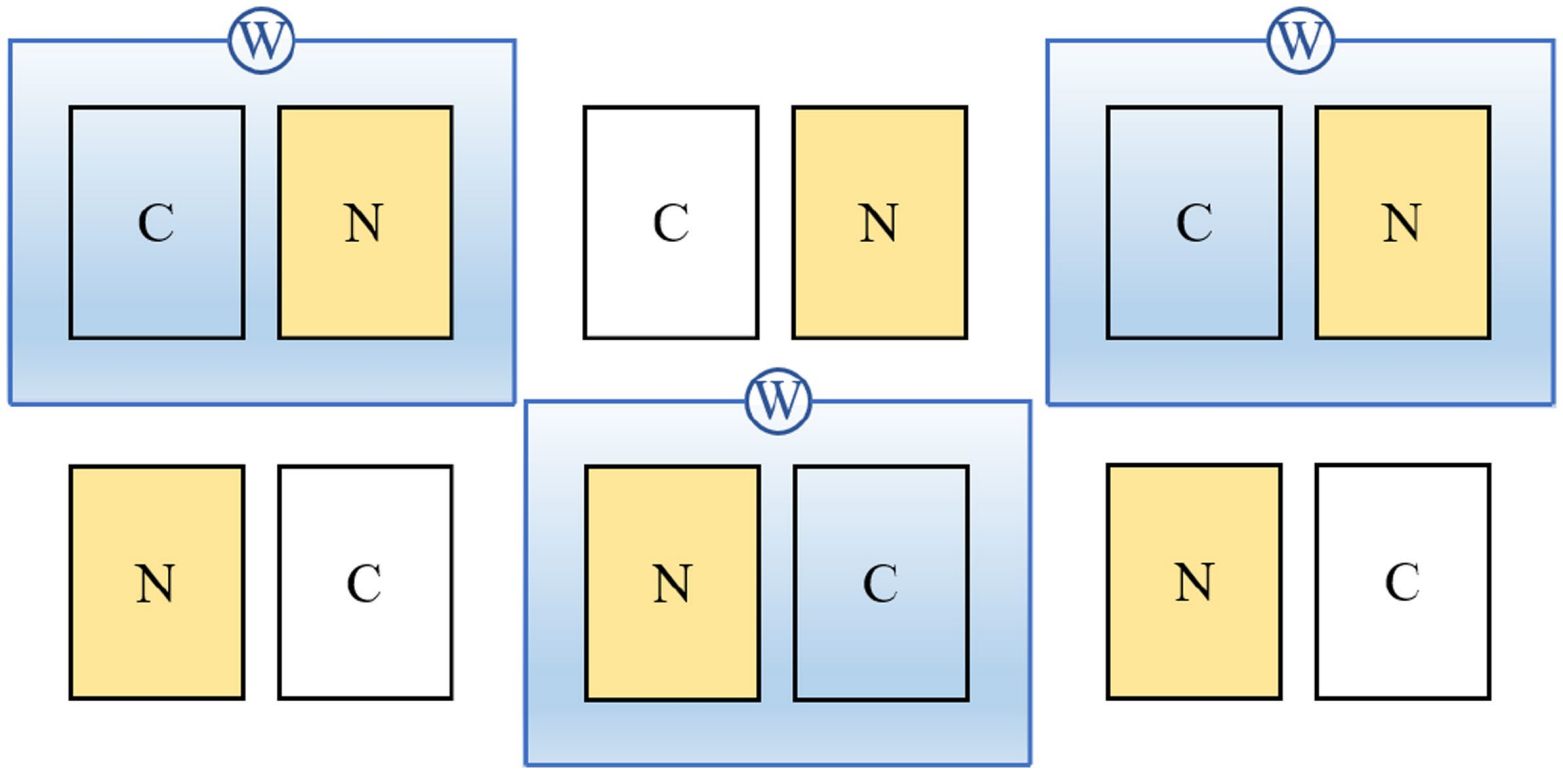
The block design of experiment treatments.

The warming and N addition experiment included three treatments: warming (1.7 ± 0.1°C, W), N addition (10 g N m^−2^ yr.^−1^, N), warming plus N addition (WN), and a control (C). There were 12 plots (3 m × 4 m), with three replicates of each treatment (Figure 1). To avoid edge effects, the experimental plots were separated by 3 m buffer strips. To generate a continuous warming environment, infrared radiation was used in the warming plots (Zhang et al., 2015). In the control and N addition plots, “dummy radiators” of the same size were used to eliminate infrared radiator shading effects. The description type and setup of the infrared radiators are described in detail by Mei et al. (2019). In the N addition plots, the experimental plot was treated using NH_4_NO_3_ (1 g N m^−2^ in 10 L water) from May to September every 2 weeks, and the total N input was 10 g N m^−2^ each year. Soil temperature and moisture were recorded by Datalogger (CR1000, Campbell Scientific Inc., Logan, UT, United States), which was inserted into the soil. The experiment started in April 2015 and the experimental plot had no other land use history, such as grazing activities.

### 2.3. Sampling and measurements

To determine the impact of warming and N addition on soil microbial community composition and structure, soil samples were collected in August 2019. Before the samples were collected, the fallen leaves and other surface organic matter were removed. The pH value of the soil, soil moisture, and the contents of soil NH_4_^+^-N, NO_3_^−^-N, total N, and available phosphorus (P) were determined. Five randomly placed soil cores (3 cm in diameter, 20 cm in depth) were drilled in each plot. These five cores from each plot were mixed. To remove debris, plant roots, and gravel, the samples were sieved (2 mm mesh) before measuring. The soil samples were stored in an ice box and shipped to the laboratory within 24 h. Each sample was divided into two parts, one for determining the general properties of the soil and the other for molecular analysis, which was stored at −80°C until further use.

The pH value of the soil in deionized water was determined with a glass electrode. Soil NH_4_^+^-N, NO_3_^−^-N, and total N were measured using a continuous flow analyzer (Mei et al., 2019). Soil available P was extracted using 0.5 mol L^−1^ NaHCO_3_, which was determined according to the methods described by Mei et al. (2019).

### 2.4. Plant community composition

In August 2019, a 1 m × 1 m subplot was randomly selected and installed in each plot to monitor plant community composition changes. The number and density of each plant species were recorded within each quadrat to calculate plant species richness (mean species number per square meter), and a square grid method was used to estimate the coverage of individual species.

### 2.5. Soil DNA extraction and Illumina HiSeq sequencing

The soil DNA was extracted by using the Power Soil DNA Isolation Kit (MO BIO Laboratories), which was quantified using a NanoDrop Spectrophotometer. Then, the extracted DNA was stored at −80°C until further processing. The V3-V4 region of the bacterial 16S rRNA gene was amplified by primers 338-F (5′-ACTCCTA CGGGAGGCAGCA-3′) and 806-R (5′-GGACTACHVGGGTWTC TAAT-3′) (Mori et al., 2014). The ITS1 region of the fungal rRNA gene was amplified using primers ITS1-F (5’-CTTGGTCATTTAGAGGA AGTAA-3′) and ITS2-R (5’-GCTGCGTTCTTCATCGATGC-3′) (Gardes and Bruns, 1993).

The amplification of polymerase chain reaction (PCR) products was extracted using the DNA Gel Extraction Kit according to the methods described by Yang et al. (2019) and Wang et al. (2019). Finally, all PCR products were quantified by Quant-iT dsDNA HS Reagent and pooled together. High-throughput sequencing analysis of bacterial and fungal rRNA genes was performed using the Illumina HiSeq 2,500 platform (2 × 250 paired ends) at Biomarker Technologies Corporation, Beijing, China.

### 2.6. Sequence analyses

The paired-end reads of the fungal ITS region amplicons and bacterial 16S rRNA gene were processed, and the ITS region and 16S sequences were screened for quality control according to the methods described earlier (Guo et al., 2019). QIIME (version 1.8.0) was used to cluster the tags >97% into operational taxonomic units (OTUs). The tags were classified into different taxonomies according to the Silva and UNITE databases for soil bacterial and fungal communities, respectively. There were 18,817 OTUs of soil bacteria and 4,646 OTUs of soil fungi after removing those OTUs that did not belong to the soil bacterial and fungal communities.

### 2.7. Data analysis

To determine the impact of warming, N addition and their interactive effects on plant species richness and coverage, the temperature of the soil, soil moisture, pH, the contents of NH_4_^+^-N, and NO_3_^−^-N in the soil, the contents of total N and available P, bacterial richness, and the species diversity and richness of the soil bacterial and fungal communities, linear mixed-effects models were performed with warming and N addition as fixed factors and block as a random factor. The interactive effects were classified according to the methods reported by Zhou et al. (2019). Additionally, the differences among the four treatments were evaluated using one-way ANOVAs with Tukey’s test. A correlation heatmap was constructed to look for relationships between soil (soil temperature and moisture, the available N concentration and pH), plant community composition (species richness and coverage), and soil microbial community.

The microbial diversity was based on the Illumina HiSeq sequencing platform, using the paired-end method to construct small fragment libraries for sequencing. By splicing and filtering reads, clustering OTUs, and performing species annotation and abundance analysis, the species composition of the sample can be revealed for further alpha diversity analysis, beta diversity analysis and significant species difference analysis, etc.

The composition and structure of soil bacterial and soil fungal communities were analyzed through permutational analyses of variance (PERMANOVA; 999 permutations) using the “vegan” package. QIIME software was used to statistically examine differences among fungal and bacterial communities across all treatments and calculate the two first components of the Nonmetric multidimensional scaling (NMDS) for fungal and bacterial communities. NMDS analysis mainly used the Bray-Curtis algorithm to calculate the distance between samples to obtain the β-value. To determine the soil and plant factors affecting soil fungal and bacterial community structure, redundancy analysis (RDA) was performed using the “vegan” package in R (version 3.5.2).

## 3. Results

### 3.1. Effects of warming and N addition on soil and plant parameters

**Figure 2:**
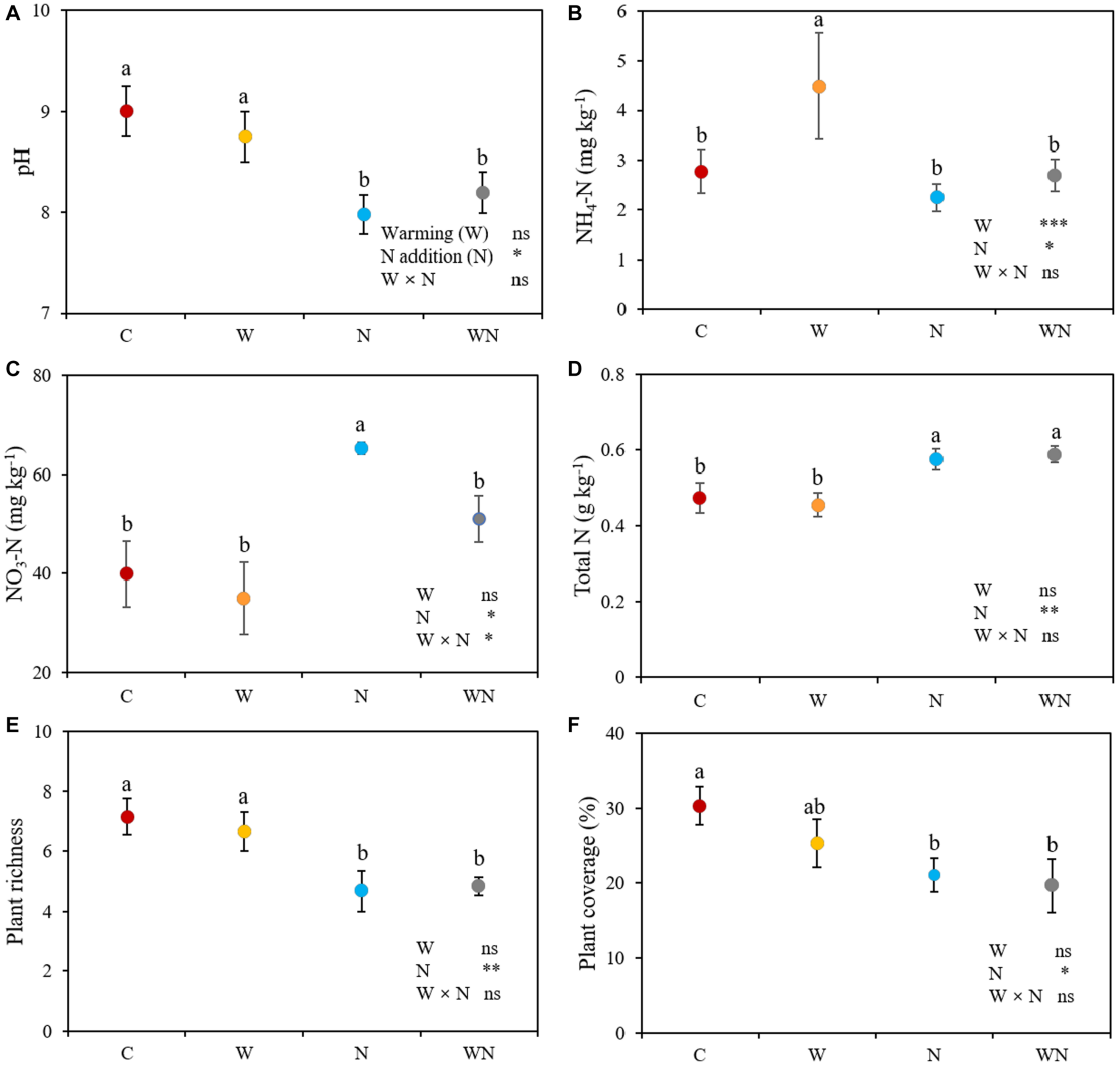
Effects of warming and nitrogen (N) additions on soil pH **(A)**, NH_4_^+^-N **(B)**, NO_3_^−^−N **(C)**, total N **(D)**, plant species richness **(E)** and coverage **(F)**. C, control; W, warming; N, nitrogen addition; WN, warming plus N addition. Lowercase letters represent significant differences at the 0.05 level. *p < 0.05; **p < 0.01; ***p < 0.001; ns represents not significant.

Warming did not affect soil pH (*p* > 0.05). Nitrogen addition and warming plus N addition reduced the soil pH by 11.4% (*p* < 0.05) and 8.9% (*p* < 0.05), respectively, compared to the control (Figure 2A). Compared with the control, warming increased the content of soil NH_4_^+^-N by 62.3% (*p* < 0.01; Figure 2B), but had no impact on NO_3_^−^-N (*p* > 0.05; Figure 2C). Nitrogen addition significantly reduced the NH_4_^+^-N by 18.7% compared with the control, but increased NO_3_^−^-N and total N concentrations by 27.5% (*p* < 0.05; Figure 2C) and 18.1% (*p* < 0.05; Figure 2D), respectively. Warming plus N addition significantly increased the NO_3_^−^-N and total N concentrations compared with the control, but had no impact on the NH_4_^+^-N concentration (*p* > 0.05). No combined effects of warming × N addition on soil pH, soil NH_4_^+^-N and total N were detected (all *p* > 0.05), and the interactive effect between warming and N addition on soil NO_3_^−^-N concentration was antagonistic (*p* < 0.05).

Warming had no effect on plant species richness (*p* > 0.05; Figure 2E) or coverage (*p* > 0.05; Figure 2F). Plant species richness and coverage in the N addition treatment was 21.1% (*p* < 0.05; Figure 2E) and 33.3% lower than that in the control (*p* < 0.05; Figure 2F). Warming plus N addition significantly reduced plant species richness (*p* < 0.05) and coverage (*p* < 0.05) compared to the control, but there was no significant difference between the N addition and warming plus N addition treatments (Figures 2E,F). There were no combined effects of warming + N addition on plant species richness (*p* > 0.05) or coverage (*p* > 0.05).

### 3.2. Effects of warming and N addition on soil fungal and bacterial α-diversity

**Figure 3:**
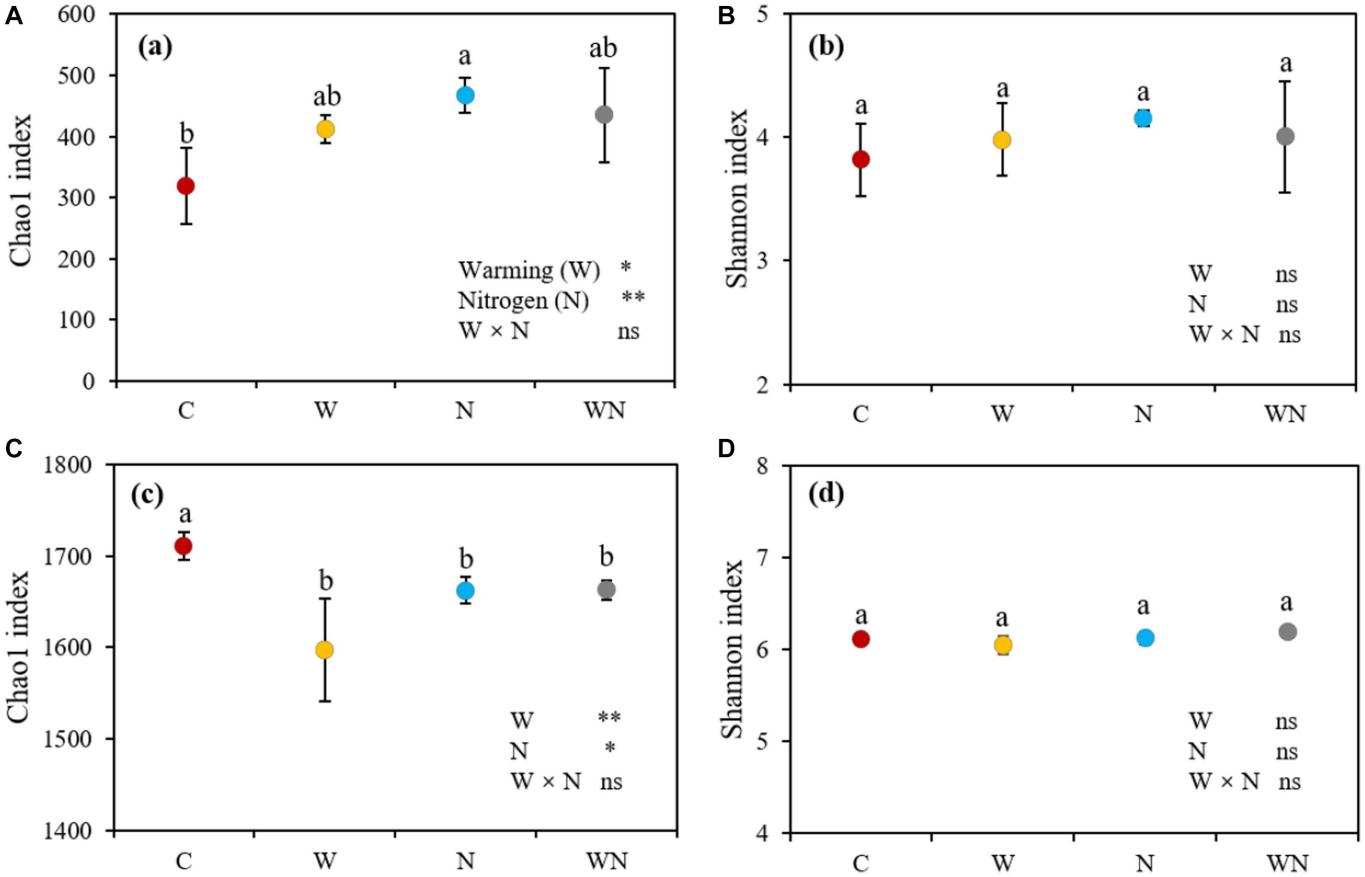
Effects of warming and nitrogen (N) additions on the soil bacterial Chao1 index **(A)** and Shannon index **(B)** and the soil fungal Chao1 index **(C)** and Shannon index **(D)**. C, control; W, warming; N, nitrogen addition; WN, warming plus N addition. Lowercase letters represent significant differences at the 0.05 level. *p < 0.05; **p < 0.01; ***p < 0.001; ns represents not significant.

Both warming and N addition treatments had significant positive effects on soil fungal α-diversity in terms of the Chao1 index (Figure 3A), warming, N addition, warming plus N addition increased the Chao1 of soil fungi by 29.4% (*p* < 0.05), 46.8% (*p* < 0.05) and 36.5% (*p* < 0.05) compared to the control, respectively. Neither warming nor N addition affected the soil fungal Shannon index (Figure 3C). No combined effects of warming + N addition on soil fungal Chao1 and Shannon indices were detected (Figure 3). Warming, N addition, and warming plus N addition decreased the Chao1 index of soil bacteria by 6.6% (*p* < 0.05), 2.7 (*p* < 0.05) and 2.8% (*p* < 0.05), respectively, compared to the control, respectively, but had no impact on the Shannon index of soil bacteria. No significant differences in the Chao1 index and Shannon index of soil bacteria among warming, N addition treatment, and warming plus N addition treatments were observed. We did not find that the combined effects of warming + N addition affected the soil bacterial Chao1 and Shannon indices (Figure 3).

### 3.3. Effects of warming and N addition on soil fungal and bacterial community compositions

**Figure 4:**
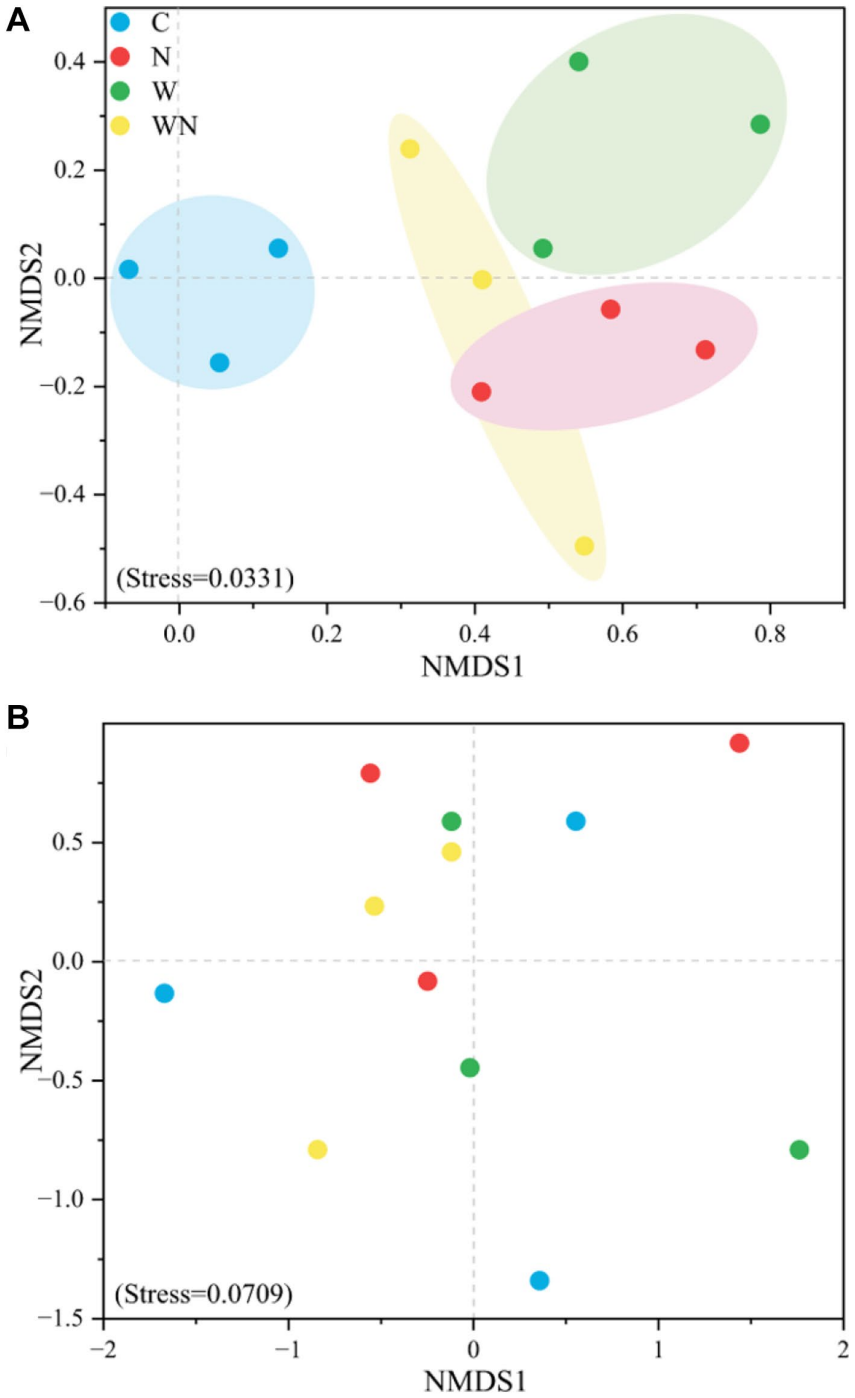
NMDS patterns of soil fungal **(A)** and bacterial **(B)** community compositions in response to warming and N addition. C, control; W, warming; N, N addition; WN, warming plus N addition.

NMDS analysis based on Bray–Curtis distance showed that the soil fungal community structure in the warming and N addition treatments was separated from that in the control (Figure 4A), while the soil bacterial community structure in neither the warming nor the N addition treatment was completely separated from that in the control samples (Figure 4B).

**Figure 5:**
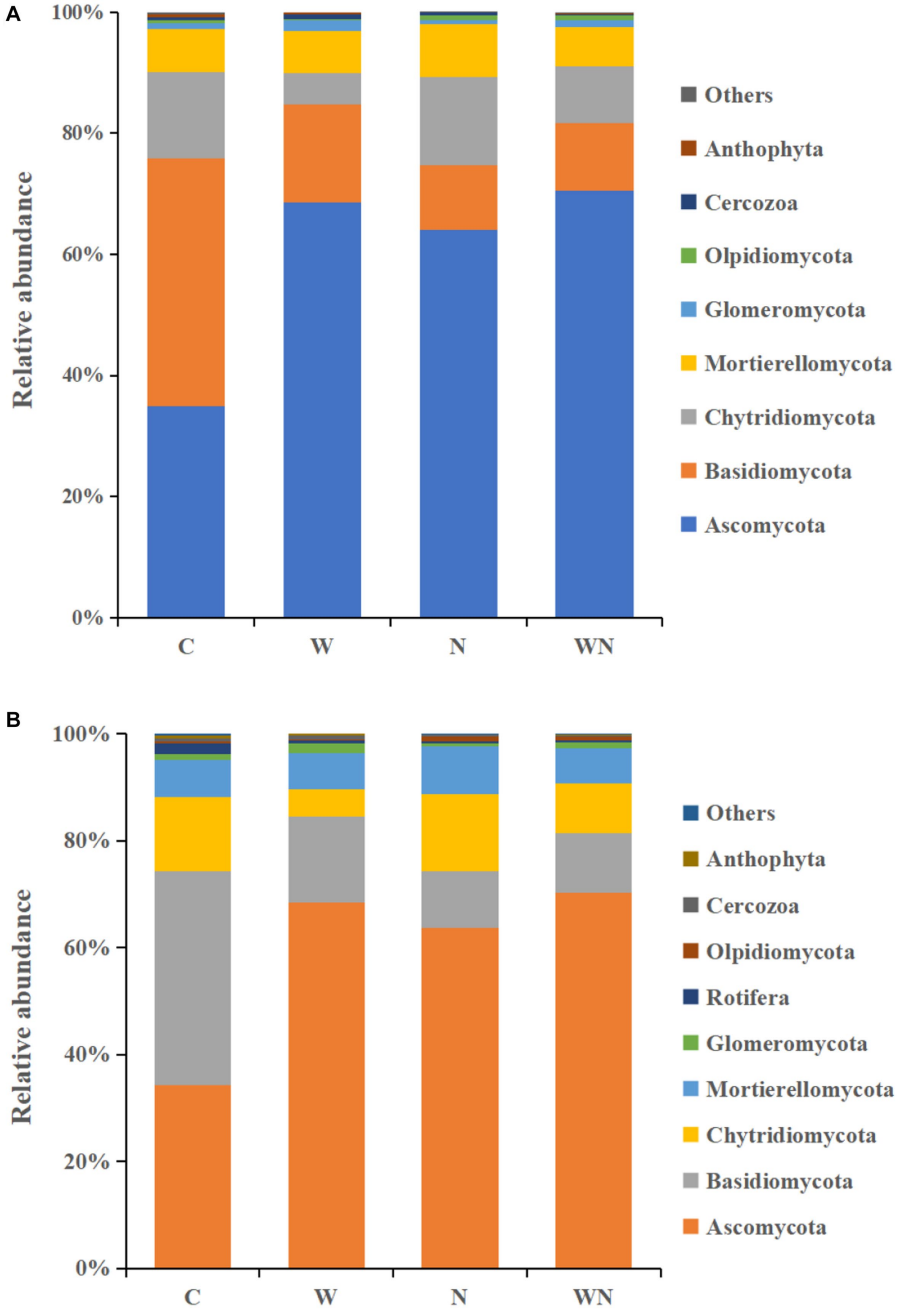
Effect of warming and N addition on the relative abundances of the soil fungal **(A)** and bacterial **(B)** groups. C, control; W, warming; N, nitrogen addition; WN, warming plus nitrogen addition. The groups with relative abundances higher than 1% are shown, while those with less than 1% relative abundance are integrated into “other.”

The soil fungal community was dominated by Ascomycota (33.2%), Basidiomycota (11.5%), Chytridiomycota (6.1%), and Mortierellomycota (4.2%) across all samples (Figure 5A). Warming significantly increased Ascomycota and Glomeromycota by 95.9 and 77.6% and decreased Basidiomycota and Chytridiomycota by 60.5% and 63.3%, respectively. Nitrogen addition significantly increased the abundances of Ascomycota and Mortierellomycota by 64.5% and 11.6%, respectively, and decreased the abundance of Basidiomycota and Glomeromycota by 76.6% and 48.6%, respectively. Proteobacteria (39.6%), Acidobacteria (18.6%), Actinobacteria (13.4%), Gemmatimonadetes (12.5%), and Chloroflexi (6.6%) dominated the soil bacterial community across treatments (Figure 5B). Warming increased the abundance of Actinobacteria, Gemmatimonadetes and Chloroflexi by 22.5%, 31.1%, and 12.2%, respectively, and reduced the abundance of Proteobacteria and Nitrospirae by 17.4% and 23.5%, respectively. Nitrogen addition did not affect the relative abundance of dominant bacterial taxa, and the interaction of warming and N addition had no impact on the abundance of dominant bacterial taxa (*p* > 0.05).

### 3.4. Relationships between soil fungal and bacterial community structure and environmental factors

**Figure 6:**
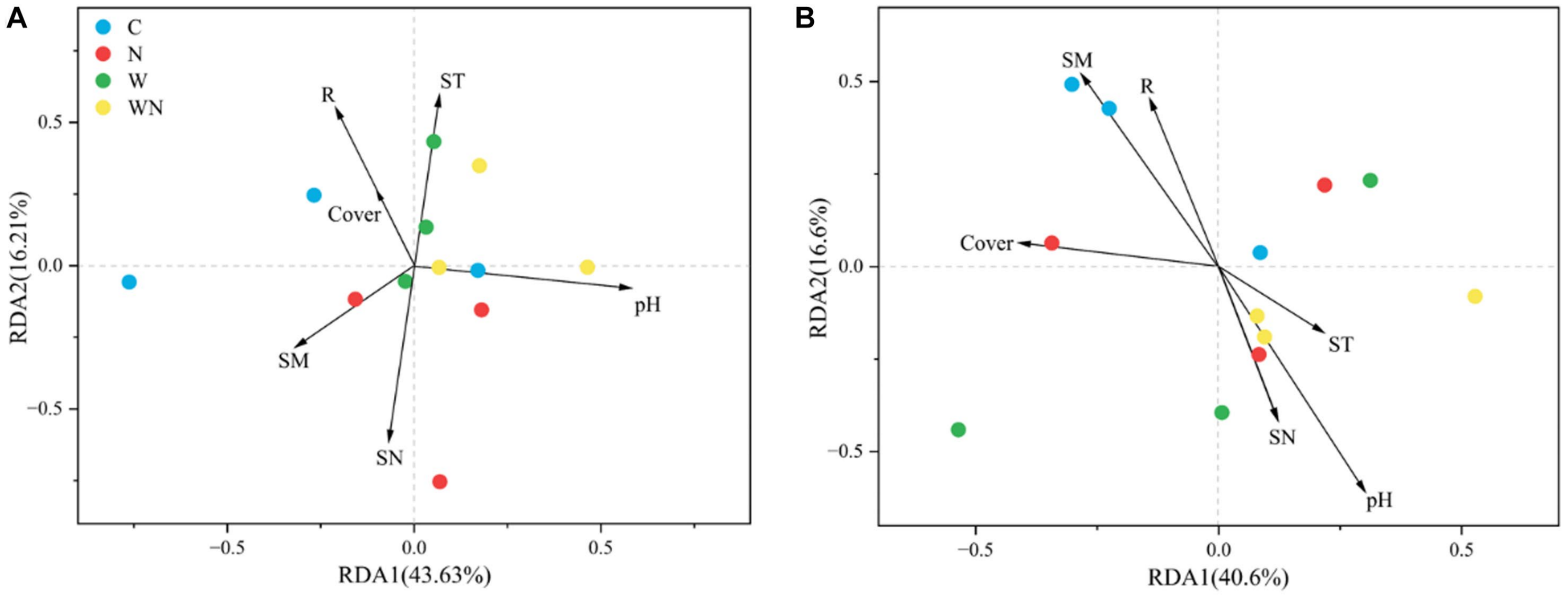
Soil fungal **(A)** and bacterial **(B)** community compositions and their variation partition ordination plots of distance-based redundancy analysis (db-RDA) under warming and N addition treatments. R, plant species richness; cover, plant coverage; ST, soil temperature; SM, soil moisture; SN, soil available N concentration; pH, soil pH.

The RDA results (Figure 6A) revealed that the soil fungal community structure was mainly affected by soil pH, soil available N (SN), soil temperature (ST, Supplementary Figure S2), soil moisture (SM, Supplementary Figure S2), and plant species richness (R). The soil bacterial community structure was mainly associated with soil pH, SM, R, and plant coverage (Figure 6B). For fungi, the correlation heatmap results showed that plant species richness was negatively correlated with the abundance of Cercozoa and Mortierellomycota. ST was positively correlated with Ascomycota and Mortierellomycota but was negatively correlated with Rotifera (Supplementary Figure S1). For soil bacteria, soil temperature was negatively correlated with Acidobacteria (Supplementary Figure S1), plant coverage was positively correlated with Acidobacteria but negatively correlated with Proteobacteria, and plant richness was only negatively correlated with Actinobacteria (Supplementary Figure S1).

## 4. Discussion

### 4.1. Effects of warming and N addition on soil fungal diversity

Notably, warming significantly increased soil fungal OTU richness and α-diversity in terms of the Chao1 index, suggesting that the 5 years of continuous warming primarily increased the number of soil fungal species in the temperate meadow. These results did not support our first hypothesis that warming would reduce soil fungal richness. Our results are not consistent with previous results obtained in a temperate desert steppe (Jia et al., 2023) and cold alpine grasslands (Qi et al., 2021) but are consistent with the results in the Northern Tibet alpine meadow (Yu CQ, et al., 2019) and in a Mediterranean shrubland (Birnbaum et al., 2019). The results suggest that warming might stimulate the growth of fungi in the lower temperature meadow ecosystem because in comparison to other species, the dominant species *Leymus chinensis* is a perennial that has a long-lived root system that responds more slowly to environmental changes and thus can retain soil fungal diversity. The stability of dominant species tends to positively affect the stability of community productivity (Yang et al., 2016; Ma et al., 2017), thereby positively affecting soil fungal diversity. Moreover, some results have shown that the response of soil fungal diversity to elevated temperature might be largely fungal functional group-specific (Geml et al., 2015; Treseder et al., 2016); for instance, warming increased the richness of wood saprotrophs and fungal pathogens in a tropical forest (Looby and Treseder, 2018).

Many results have demonstrated that the influence of N addition on soil fungal diversity depends on the type of ecosystem (N-limited or N-rich) and the dose of N addition (Zhou et al., 2016). This current study found that N addition highly increased the soil fungal diversity, which is consistent with the results of previous studies in N-limited ecosystems (Mueller et al., 2014), suggesting that a small amount of N addition might have a positive effect on the growth of soil fungal species in N-limited ecosystems. The results support our previous inference that the Songnen meadow is an N-limited ecosystem based on the aboveground community composition (Zhang et al., 2015). The increase in soil fungal diversity may reduce the negative impact of N addition on plant species richness because many studies have found that the addition of N will reduce plant species diversity (Bobbink et al., 2010; Zhao et al., 2019). However, N addition is still increasing in China (Yu G, et al., 2019), so the influence of long-term N addition on soil fungal diversity is still uncertain and should be studied over a long period.

### 4.2. Effects of warming and N addition on soil bacterial diversity

Previous results from a Tibetan alpine meadow showed that warming increased soil bacterial diversity (Li et al., 2016; Yu CQ, et al., 2019), but other studies found that warming did not affect soil bacterial diversity (Zi et al., 2018; Qi et al., 2021). In the present study, warming significantly reduced the diversity of soil bacteria, which is consistent with a previous result from a desert steppe in northern China (Jia et al., 2023). The results suggest that the response of soil bacteria is not consistent. First, the possible reason for the decrease in soil bacterial diversity might be related to the slight decrease in the soil N concentration caused by warming. Second, the decrease in soil bacteria might be explained by the slight decrease in soil pH (Figure 2) because the decline in pH could reduce the richness of soil bacteria (Wang et al., 2018). Third, the difference in soil moisture caused by precipitation might partly explain this inconsistency because warming could affect soil moisture. Moreover, the results indicate that the response of soil bacterial diversity to global warming is faster than that of aboveground communities.

In this study, N addition reduced the α-diversity of soil bacteria, which is consistent with previous results in tropical forest, temperate steppe and arctic tundra ecosystems (Campbell et al., 2010; Zeng et al., 2016; Wang et al., 2018), which supports our first hypothesis that N addition reduces soil bacterial diversity. This might be explained partly by the decline in plant species richness and coverage induced by N addition (Zhao et al., 2019). Although several studies have found that N addition reduces soil bacterial diversity by decreasing soil pH, in the present study, N addition had no effect on soil pH, suggesting that the influence of N addition on soil bacterial diversity might depend on the ecosystem type. This result suggests that the changes in soil pH might not be the main factors affecting soil bacterial diversity in the soda-saline meadow because of the high pH, which cannot easily decline due to N addition. The decline in soil bacterial diversity might alter other soil ecological functions, such as nitrification and denitrification, which will affect N loss in this meadow ecosystem. However, in the present study, we did not focus on these aspects, so the impact of soil bacterial diversity caused by N addition on the other ecological functionalities should be further studied.

### 4.3. Effects of warming and N addition on the composition and structure of soil fungal communities

Some studies have found that warming has a significant impact on the soil fungal community structures in several ecosystems (Looby and Treseder, 2018; Che et al., 2019); however, several studies have found that short-term warming does not affect the structure of the soil fungal communities in alpine grasslands (Hayden et al., 2012 Zhang T, et al., 2016). In the present study, warming significantly altered the soil fungal community structure (Figure 5) and increased the relative abundance of Ascomycota, which might explain the soil carbon (C) loss caused by global warming because Ascomycota is a saprotrophic fungus that plays an important role in soil organic carbon decomposition (Xiong et al., 2014). In addition, warming also increased Glomeromycota abundance (arbuscular mycorrhizal fungi), which can also stimulate soil organic matter decomposition (Kang et al., 2020). Moreover, the RDA results suggest that warming could alter the structure of soil fungal communities by affecting soil properties (pH, soil nitrogen concentration, and soil temperature) rather than by affecting plant community composition, suggesting that the response of soil fungal community structure to warming might be more rapid than that of a plant community.

Previous results have demonstrated that N addition alters the structure of soil fungal communities (Leff et al., 2015). N addition significantly increased Ascomycota abundance in the studied ecosystem. The possible reason for the increase in Ascomycota might be related to the imbalance in C:N caused by N addition because the increase in Ascomycota caused by N addition might enhance soil C decomposition. The decline in Basidiomycota abundance might reduce the competitive pressure of Ascomycota for resources (Weber et al., 2013). In addition, N addition significantly reduced Glomeromycota abundance, which supported our previous results that N addition reduced the competition of mycorrhizal plant species in the Songnen meadow ecosystem (Zhang T, et al., 2016).

### 4.4. Effect of warming and N addition on the composition and structure of soil bacterial communities

Previous studies have shown that warming could alter the soil bacterial community structure because of the difference in bacterial species that respond to warming (Zhang KP, et al., 2016). In this study, warming increased the relative abundances of Actinobacteria, Gemmatimonadetes and Chloroflexi, and the increase in Actinobacteria and Chloroflexi might be related to the increase in soil CO_2_ efflux caused by warming because Actinobacteria might enhance soil organic matter decomposition. Moreover, the increase in Gemmatimonadetes might increase soil nitrification and affect soil N cycling. Several studies have shown that a decline in pH can affect soil bacterial community composition (Wang et al., 2018). In the current study, warming slightly decreased the soil pH, which might have been due to the increase in NH_4_^+^-N caused by warming (Figure 2). In addition, the increase in Actinobacteria might reduce the negative influence of warming on plant growth because Actinobacteria could improve plant growth by reducing soil disease suppression (Palaniyandi et al., 2013). The relationship between soil bacterial community structure and plant disease infection under warming should be studied in the future, which might be helpful to understand the mechanism of how belowground community composition affects aboveground community composition.

Many studies have shown that N addition plays a key role in affecting soil bacterial community structure (Ramirez et al., 2012); nevertheless, the response of soil bacterial community composition to N addition is not consistent in grasslands across the globe (Leff et al., 2015). The present results found that N addition had no impact on soil bacterial community composition or did not affect the abundance of the dominant bacterial phylum. The possible reason is that N addition had no effect on soil pH (Wang et al., 2018; Guo et al., 2019). The results suggest that the response of the soil bacterial community structure to N addition is lower than that of plant communities (Zhao et al., 2019), and plant species richness and cover caused by N addition had few effects on the soil bacterial community structure (Figure 6). However, an increase in Chloroflexi abundance and a decline in Nitrospirae abundance were also detected, which might have affected N cycling because Chloroflexi and Nitrospirae are related to N fixation and nitrification (Pedrinho et al., 2020).

## 5. Conclusion

Our results showed that simulated warming and N addition significantly increased soil fungal species diversity and altered fungal community structure in the studied temperate meadow ecosystem because of the increase in ammoniation caused by warming and the increase in nitrification caused by the addition of nitrogen. Warming and N addition significantly reduced soil bacterial diversity but had few impacts on the structure of the soil bacterial community. Warming and N addition did not interactively affect the compositions of the soil fungal and bacterial communities. Changes in plant species richness play a vital role in the soil fungal community structure, while changes in plant cover play an important role in the soil bacterial community structure. Our results highlight that the resistance of the soil fungal community structure to global warming and N addition might be lower than that of the soil bacterial community structure, and the influence of global changes on the soil fungal and bacterial community structures by affecting plant community composition might not always be consistent. Moreover, the present results suggest that the influence of global changes on the soil microbial community might be determined, to some extent, by the plant community, soil physicochemical properties, and climate characteristics at the regional scale.

## Data availability statement

The datasets presented in this study can be found in online repositories. The names of the repository/repositories and accession number(s) can be found at: https://www.ncbi.nlm.nih.gov/genbank/, PRJNA686700.

## Author contributions

TZ and JG conceived the ideas and designed methodology. MJ and YT collected and analyzed the data. MJ and TZ wrote the first draft of the manuscript. RG, SL, and JG revised the manuscript. MJ, YT, RG, SL, JG, and TZ contributed critically to the drafts and gave final approval for publication. All authors contributed to the article and approved the submitted version.

## Funding

This research was funded by the National Natural Science Foundation of China (32171645), Foundation of Science and Technology Commission of Jilin Province (20200201115JC), the Fundamental Research Funds for the Central Universities (2412020ZD010), and the Program of Introducing Talents of Discipline to Universities (B16011).

## Conflict of interest

The authors declare that the research was conducted in the absence of any commercial or financial relationships that could be construed as a potential conflict of interest.

## Publisher’s note

All claims expressed in this article are solely those of the authors and do not necessarily represent those of their affiliated organizations, or those of the publisher, the editors and the reviewers. Any product that may be evaluated in this article, or claim that may be made by its manufacturer, is not guaranteed or endorsed by the publisher.
