# Supplementary material for: Effects of warming and nitrogen addition on soil fungal and bacterial community structures in a temperate meadow
Source: Front Microbiol. 2023 Jul 12;14:1231442. doi: 10.3389/fmicb.2023.1231442 (PMC10369075; doi:10.3389/fmicb.2023.1231442)
Supplement: Supplementary file 1 [file Data_Sheet_1.docx]

**Supplementary material**


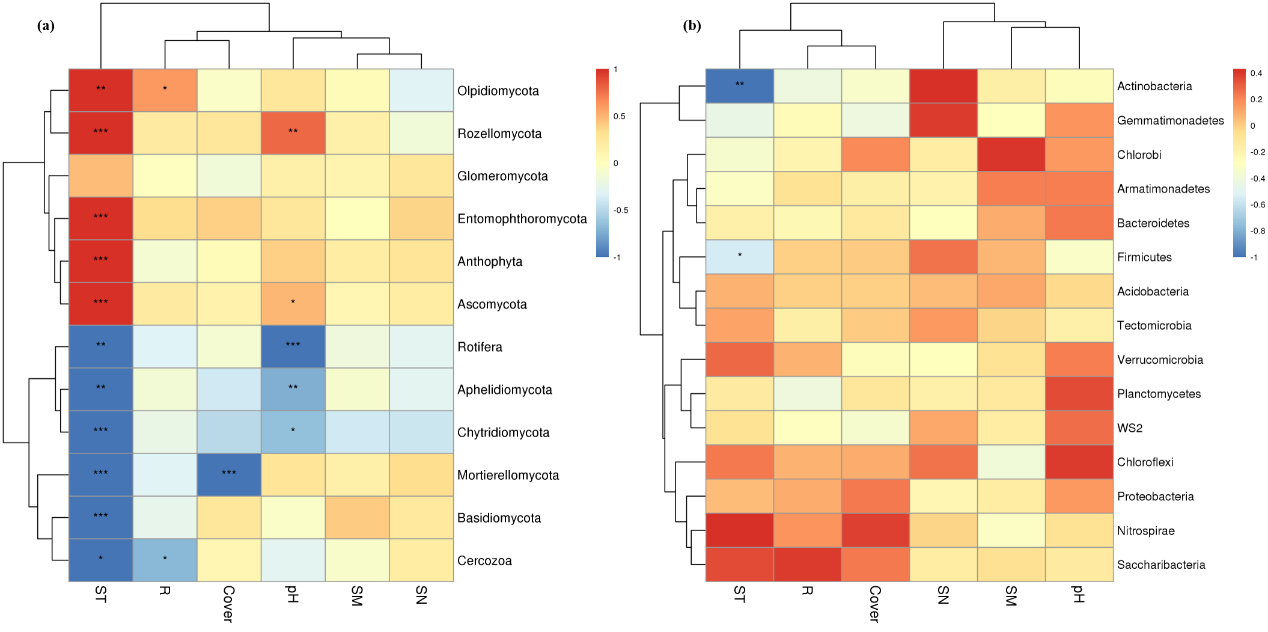


**Fig. S1** The results of correlation heatmap of soil fungal (a) and bacterial (b) community structures to soil factors and plant species richness and cover at phylum level. ST, soil temperature; R, plant species richness; Cover, plant coverage; pH, soil pH; SM, soil moisture; SN, soil available N concentration.


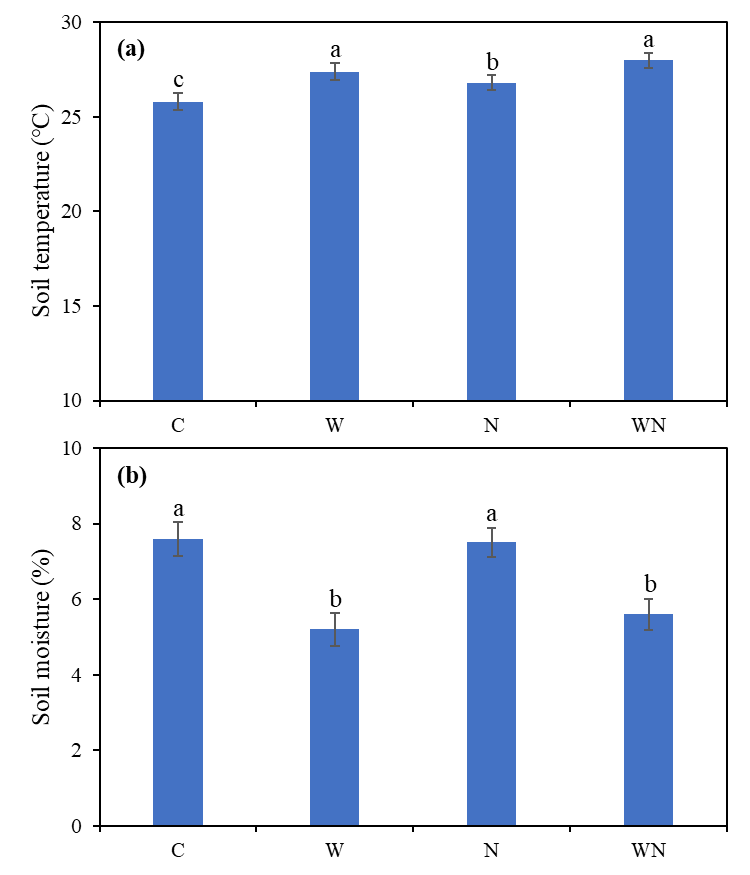


**Fig. S2** The effect of warming and N addition on soil temperature (a) and moisture (b). C, control; W, warming; N, nitrogen addition; WN, warming plus N addition.
